# Responses to Oxidative and Heavy Metal Stresses in Cyanobacteria: Recent Advances

**DOI:** 10.3390/ijms16010871

**Published:** 2014-12-31

**Authors:** Corinne Cassier-Chauvat, Franck Chauvat

**Affiliations:** UMR8221, CEA, CNRS, Université Paris-Sud, Institut de Biologie et Technologie Saclay, Laboratoire de Biologie et Biotechnologie des Cyanobactéries, CEA-Saclay, Gif sur Yvette 91190, France; E-Mail: corinne.cassier-chauvat@cea.fr

**Keywords:** heavy metals, oxidative stress, glutathione, regulation, exopolysaccharides, transport systems, *Synechocystis*

## Abstract

Cyanobacteria, the only known prokaryotes that perform oxygen-evolving photosynthesis, are receiving strong attention in basic and applied research. In using solar energy, water, CO_2_ and mineral salts to produce a large amount of biomass for the food chain, cyanobacteria constitute the first biological barrier against the entry of toxics into the food chain. In addition, cyanobacteria have the potential for the solar-driven carbon-neutral production of biofuels. However, cyanobacteria are often challenged by toxic reactive oxygen species generated under intense illumination, *i.e.*, when their production of photosynthetic electrons exceeds what they need for the assimilation of inorganic nutrients. Furthermore, in requiring high amounts of various metals for growth, cyanobacteria are also frequently affected by drastic changes in metal availabilities. They are often challenged by heavy metals, which are increasingly spread out in the environment through human activities, and constitute persistent pollutants because they cannot be degraded. Consequently, it is important to analyze the protection against oxidative and metal stresses in cyanobacteria because these ancient organisms have developed most of these processes, a large number of which have been conserved during evolution. This review summarizes what is known regarding these mechanisms, emphasizing on their crosstalk.

## 1. Introduction

Cyanobacteria, the ancient prokaryotes that perform oxygen-evolving photosynthesis, are viewed as the producers of the Earth’s oxygenic atmosphere [1]; and the ancestors of plant chloroplasts [2]. Contemporary cyanobacteria continue to play a crucial role in biogeochemical cycles in fixing about 25 Giga tons of carbon from CO_2_ per year into energy dense biomass [3]. Hence, cyanobacteria are regarded as a promising “low-cost” microbial factory for CO_2_ capture and storage and the ecologically responsible production of biofuels, in allowing to save arable soils, pure fresh water, fertilizers and pesticides for agriculture [4,5]. This is especially true in the case of the unicellular model strain *Synechocystis* PCC6803, which has a small genome [6] and is easily manipulated [7,8,9,10,11]. Such powerful genetics is important because natural cyanobacteria miss some of the required biofuel producing enzymes.

In colonizing most waters (fresh and marine) and soils, cyanobacteria have evolved as the most diverse groups of bacteria [12,13]. As a consequence, the genome of cyanobacteria is widely diverse in size and GC% (ranging from 30% to 60%) [14], probably as the results of gain-and-loss of genes transferred by plasmids, insertion sequences and/or phages. Most cyanobacteria possess a single circular chromosome ranging from about 1.4 Mbp to about 9.0 Mbp in size. In addition, many cyanobacteria possess plasmids (a few Kbp to several hundreds of Kbp in size). For instance, *Synechocystis* PCC6803 possesses seven plasmids, ranging from 2.3 Kbp [15] to 119 Kbp [6]. By contrast, a few marine cyanobacteria (*Prochlorococcus* and *Synechococcus*) have no plasmids, whereas *Cyanothece* ATCC51142 possesses two chromosomes (one circular, 4.9 Mbp; and one linear, 0.4 Mbp) and four plasmids (ranging from 10 to 39 Kbp). Furthermore, cyanobacteria display different cell morphologies (spherical or cylindrical) and forms (unicellular or multi-cellular filaments, some of which being capable of fixing atmospheric nitrogen). Hence, cyanobacteria are attractive models to study the influence of the environment on the physiology, metabolism, morphology, division and differentiation of microbial cells [14,16,17,18,19,20].

**Figure 1:**
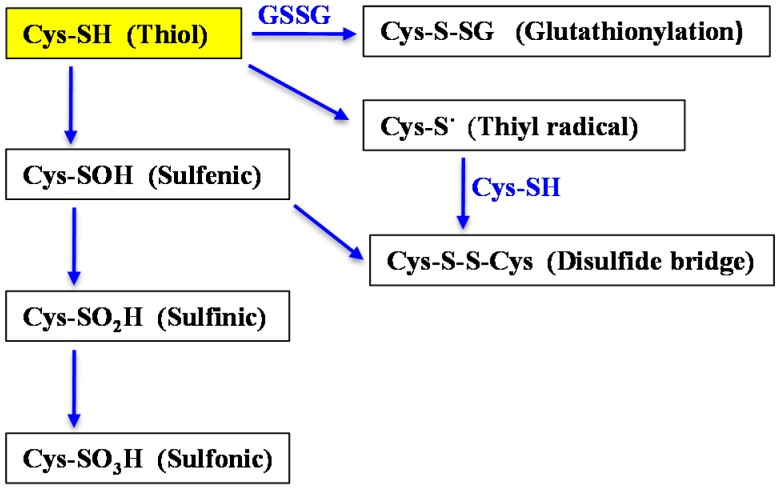
Schematic representation of the processes involved in metal homeostasis and detoxification (see text for abbreviations). The normal (reduced monomer) and oxidized (disulfide, dimer) forms of glutathione are represented by GSH (the reduced form of gluthatione) and GSSG (the oxidized form of glutathione), respectively. The blue arrows point into the direction of oxidation.

Because of their photoautotrophic lifestyle, cyanobacteria are inevitably challenged with toxic reactive oxygen species (ROS) produced by their metal-rich photosynthetic apparatus [17,21]. These oxidative agents, singlet oxygen (^1^O_2_), the superoxide anion (O_2_^·−^), hydrogen peroxide (H_2_O_2_), and hydroxyl radical (OH) can oxidize the thiol of the cysteine residues of proteins (–SH) into sulfenic (–SOH), disulfides (–S–S–), sulfinic acids (–SO_2_H) or sulfonic acids (–SO_3_H) [22]. The disulfide bridges can link two cysteinyl residues from the same or different proteins; or from a protein and a molecule of the anti-oxidant tripeptide glutathione (γ-l-glutamyl-l-cysteinyl-l-glycine). The formation of the later glutathione-protein mix disulfide, also termed glutathionylation, is regarded as a transient protection of critical cysteines against irreversible oxidation (formation of sulfinic and sulfonic acids) during oxidative stress and/or as a post-translational regulatory modification (Figure 1) [23,24,25].

The ROS oxidants can be detoxified by various metabolites (ascorbate, carotenoids, glutathione, vitamins, *etc.*), and the enzymes superoxide dismutase (SOD), catalase (KatG) and peroxiredoxins (Prx), which sequentially convert the superoxide anion to hydrogen peroxide (SOD) and subsequently hydrogen peroxide to water [14]. By contrast, the protein disulfides and glutathione-protein mix disulfides are repaired by thioredoxins and glutaredoxins enzymes (see below).

Like in other organisms where it has been estimated that one-quarter to one-third of all proteins require metals [26], cyanobacteria have high requirements for metal ions. This is especially true for iron (Fe), which serves in photosynthetic and other electron transfer proteins [27]. Cyanobacteria also use Molybdenum (Mo) in sulfite oxidases, nitrogenases and nitrate reductases; magnesiusm (Mg) in chlorophyll, ATPases and kinases; copper (Cu) in cytochrome oxidase and plastocyanin; manganese (Mn) in the oxygen-evolving photosynthetic complex; nickel (Ni) in hydrogenase and urease; and zinc (Zn) in RNA and DNA polymerases and in CO_2_ assimilation proteins [26,28]. Consequently, cyanobacteria have evolved elaborate mechanisms to acquire sufficient metal atoms to meet their needs and to adjust them to match supply.

In addition, cyanobacteria are also frequently challenged by heavy metals, such as aluminum (Al), arsenic (As), cadmium (Cd), cesium (Cs), chromate (Cr), mercury (Hg), lead (Pb), or uranium (U), which normally have no function as nutrients [29]. The accumulation in soils and waters of heavy metals released by natural sources (volcanoes or forest fires) and anthropogenic activities (mining, burning fossil fuels, *etc.*) have produced severe environmental contaminations in many parts of the world due to the persistence of metals in the environment and their accumulation throughout the food chain [26,30,31]. The toxicity of these metals is based on their chemical properties, which allow them to promote the production of reactive oxygen species (ROS); the inactivation of enzymes, basically by reaction with SH-groups; and/or the displacement of the normal metal cofactors of numerous metalloproteins [26,32,33].

**Figure 2:**
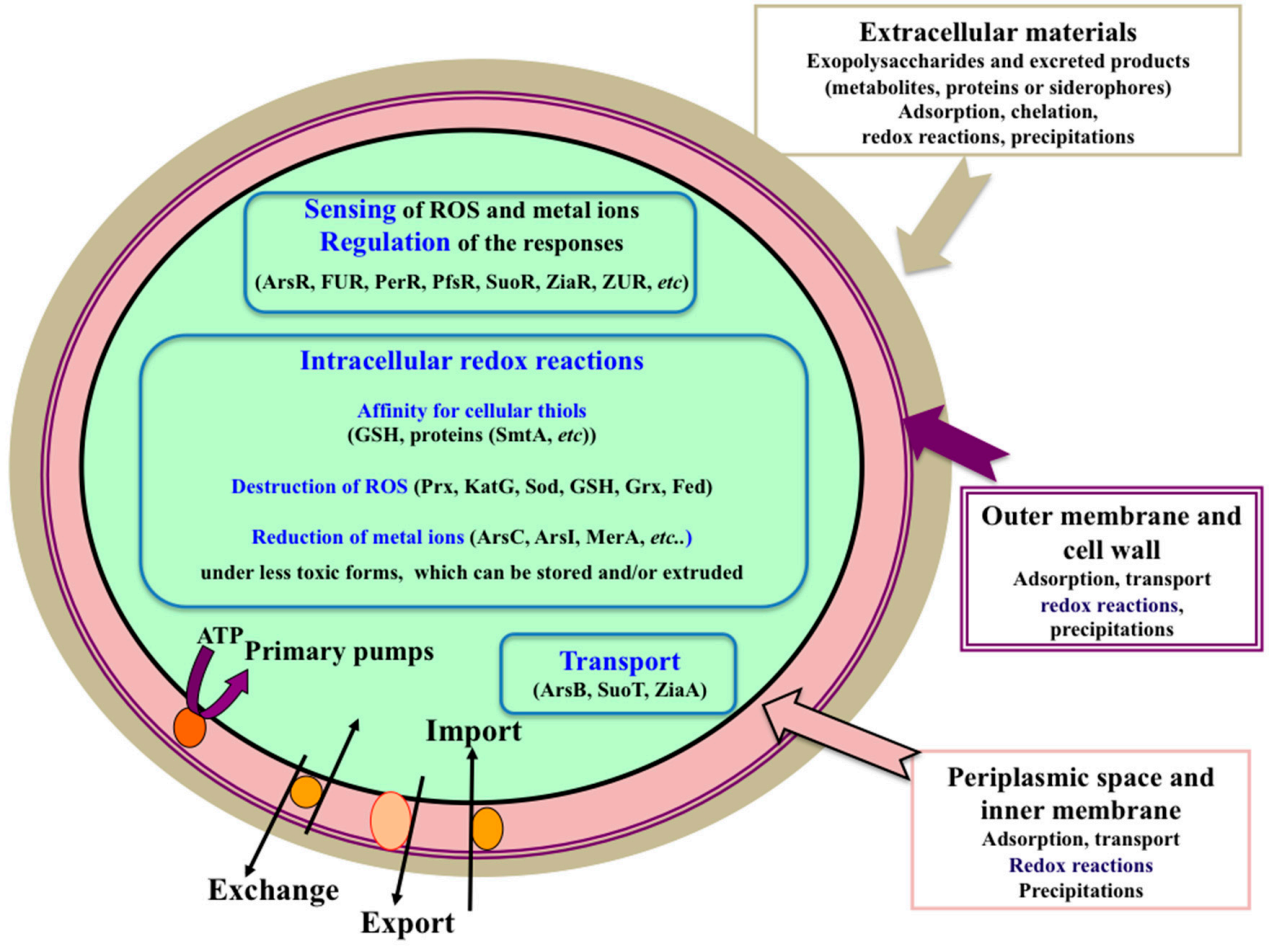
Schematic representation of the processes involved in metal homeostasis and detoxification (see text for abbreviations).

This review presents the current knowledge of the responses of cyanobacteria to heavy metals: transport, toxicity, perception and regulation of these processes; and their crosstalk with the defenses against oxidative stress (Figure 2).

## 2. The Extracellular Mantle of Exopolysaccharides Constitutes the First Protective Barrier against Metal Stresses (Cd, Co, Fe, and CeO_2_ and TiO_2_ Nanoparticles)

**Figure 3:**
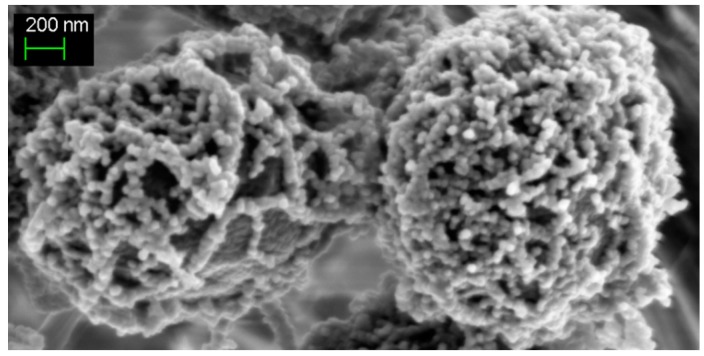
Typical SEM (scanning electron microscopy) images of the unicellular spherical-celled cyanobacterium *Synechocystis* PCC6803 showing the thick mantle of exopolysaccharides wrapping wild-type cells.

A large number of bacteria synthesize extracellular polymeric substances, mainly of polysaccharidic nature (exopolysaccharides (EPS)). These EPS serve as the structural scaffold for the formation and maintenance of biofilms, which offers a protective shielding that dramatically increases bacterial resistance to antimicrobial agents [34]. In cyanobacteria, EPS are regarded as being involved in the biomineralization of calcium (and/or magnesium) carbonates, which can generate stromatolites [3]. In addition, the presence of negative charges (uronic acids) in cyanobacterial EPS was proposed to play an important role in the sequestration of metal cations, a phenomenon of possible interest in water treatment [35]. As little was known about the production of exopolysaccharides (EPS) in cyanobacteria, we recently studied four presumptive EPS production genes in *Synechocystis*, which produces copious amounts of EPS attached to cells (CPS) and released in the culture medium (RPS) (Figure 3) [36].

These four genes designated as *sll0923*, *sll1581*, *slr1875* and *sll5052* in cyanobase [6] are dispensable to photoautotrophic growth. Three of them *sll0923*, *sll1581* and *slr1875* indeed operate in the production of both CPS and RPS. The mutant doubly deleted for *sll1581* and *slr1875* lacks the negatively charged EPS mantle that normally surrounds wild-type (WT) cells [36,37]. This EPS mantle sorbs iron atoms, which can be subsequently released and taken up by the cells when required, thereby protecting them from iron-starvation [36], a frequently encountered environmental stress [27]. We also showed that the EPS protect *Synechocystis* against the toxicity of the heavy metals cadmium and cobalt [36]. Together, these data are consistent with the earlier findings that (i) both Cd and Co disturb Fe homeostasis; and (ii) increasing Fe availability can increase the tolerance to Cd and Co [38,39]. Similarly, we found that the EPS shield *Synechocystis* from direct contacts with the oxidative stress-generating CeO_2_ [40] and TiO_2_ nanoparticles [41], which are intensively used by human industries [42].

## 3. Metallothionein and/or Efflux ATPases Operate in Zinc Homeostasis and Cadmium Tolerance

Metallothioneins (MT) are small cysteine-rich proteins, which have the capacity to bind metals (such as As, Cd, Cu, Hg and Zn) through the thiol group of its cysteine aminoacids. In cyanobacteria, MTs were first identified in cells adapted to growth in elevated levels of Cd or Zn [43,44]. In *Synechococcus* PCC7942, the cysteine-rich, Zn- and Cd-binding metallothionein is encoded by the *smtA* gene, which is divergently transcribed from *smtB*, which codes for the metal-responsive repressor of *smtA*. Elevated concentrations of the ionic species of Cd, Co, Cr, Cu, Hg, Ni, Pb and Zn elicited an increase in the abundance of *smtA* transcripts [45,46]. SmtA is not present in *Synechocystis* PCC6803, where it is replaced by an efflux ATPase (ZiaA, Slr0798 in cyanobase [6]). The *ziaA* gene is regulated by the Zn-responsive repressor ZiaR (Sll0792), which shares sequence homology with SmtB. The *ziaR* gene is transcribed divergently from *ziaA* [47], and both *ziaA* and *ziaR* belong to a gene cluster involved in sensing and homeostasis of Ni, Co and Zn [48]. Similarly to *Synechocystis* PCC6803 and *Synechococcus* PCC7942, a large number of other cyanobacteria have either a metallothionein (MT) or an efflux pump, though other species, such as various *Anabaena* and *Oscillatoria* strains, have genes for both MT and efflux pump [44]. In *Synechocystis* PCC6803, *ziaA* and the Mn transporter gene *mntC* (*sll1598*) were found to be regulated also by Sll0649, which operates in the protection against stresses triggers by Cd as well as high-concentration of Cu, Fe, Mn and Zn [49].

## 4. Transport Systems and Redox Enzymes Operate in the Defense against Arsenic

In Gram-negative bacteria, such as cyanobacteria, metals probably diffuse freely across the outer membrane through porins, while energy-coupled importers allow acquisition of metal-metabolite complexes that are too large for porins, such as Fe-siderophore complexes [26]. In addition, metals can be exported out of the cells. Hence, the number of atoms of each metal in a cell is a function of net influx and efflux, which is mediated by specific metal importers and exporters.

Arsenic can occur in different inorganic forms, such as trivalent As(III) in AsO_3_ (arsenite) or pentavalent As(V) in AsO_4_ (arsenate), depending on the redox potential of the environment. Arsenite can donate electrons to various acceptors, and this feature can be utilized in organisms performing primordial anoxygenic photosynthesis or anaerobic respiration [50,51]. On the other hand, arsenite may bind to thiol groups of proteins, thereby altering their activity, or to the antioxidant glutathione, thereby depleting its pool and contributing to ROS generation. As an analogue of phosphate, arsenate may impair biochemical reactions, such as oxidative phosphorylation and glycolysis.

As resistance systems consist in the reduction of arsenate to arsenite, which is subsequently exported outside the cell. Arsenate reduction to arsenite is catalyzed by arsenate reductase enzymes, which define at least three nonrelated protein families. These reductases use the thioredoxin, glutaredoxin or mycoredoxin systems as electron donors. Arsenite export is mediated by two families of proteins: ArsB proteins, which are present only in bacteria, and Acr3 proteins, which are more widely distributed in bacteria, fungi and plants [51]. As can also be detoxified by the widely-conserved As methylation system, which conjugates As to methyl groups thereby forming As volatile species [51].

*Synechocystis* is rather tolerant to arsenate and even better to arsenite, likely because the cells possess several As resistance systems. The main system is encoded by the *arsBHC* tricistronic operon (*slr0944*–*slr0945*–*slr0946*), which is regulated by the repressor encoded by the unlinked *arsR* gene (*sll1957*) [52]. The ArsB protein is an Acr3-like arsenite transporter, while ArsH has a FMN-quinone reductase activity with no clear function in As resistance [51,52]. ArsC is an arsenate reductase, which uses the glutathione/glutaredoxin system for reduction [24,53,54,55], and operates in Cd resistance by an as yet unknown mechanism [38].

*Synechocystis* has a second arsenate reductase, ArsI, encoded by the two nearly identical genes *arsI1* (*sll5104*) and *arsI2* (*sll6037*) [55], which is less important than ArsC for As resistance [55]. Another gene *ssarsM* (*slr0303*) is likely important for *Synechocystis* protection against As, since its expression in *E. coli* confers an increased As tolerance, and the ArsM enzyme can uses *S*-adenosyl methionine and glutathione as methyl donors, to methylate arsenite to the volatile trimethylarsine [56].

*Synechocystis* possesses another arsenic resistance system. It is encoded by the two divergently transcribed gene clusters *suoS* (*sll5036*)–*suoR* (*sll5035*) and *suoC* (*slr5037*)–*suoT* (*slr5038*), which are propagated on one of the large plasmid, but do not consitute a single operon *suoRSCT*, unlike what is improperly written in the publication [50]. The ArsR-like regulator SuoR binds to the promoter region shared by the two genes pairs, *suoS*–*suoR* and *suoC*–*suoT*, and negatively regulates their transcription. SuoS behaves as a type I sulfide:quinone oxidoreductase, while SuoT operates in arsenite transport, and SuoC has no known function.

Recently, it was shown that arsenate and arsenite trigger similar genome-wide transcriptional responses, which include induction of the redox scavenging system and chaperones, and repression of photosynthesis and growth related genes [51].

## 5. The Mercuric Reductase-Like Enzyme Plays a Prominent Role in the Reduction of, and Protection against, both Mercury and Uranium

Mercury (Hg) and uranium (U) are highly disseminated in natural waters by human activities, such as mining, dentistry, use of fungicides in papermaking and agriculture (Hg), or in production of nuclear energy (U) [26,32,57]. Hg is emitted primarily as volatile elemental (Hg0), which can travel long distances through the atmosphere, before settling in terrestrial or aquatic ecosystems (for instance the artic is regarded as a sink for mercury [31]). Hg0 can be oxidized by anaerobic bacteria to HgII, which can enter in photoautotrophs and poison photosynthesis and the aquatic food chains [32]. HgII can be detoxified by photoautotrophs, including cyanobacteria, through transformation into meta-cinnabar (β-HgS) [58] or reduction to Hg0 by the mercuric reductase enzyme [32]. To study how *Synechocystis* PCC6803 protects itself from HgII we analyzed its *slr1849* gene, which encodes a protein resembling the bacterial mercuric reductase MerA enzyme, which is widely distributed in cyanobacteria. As anticipated, we found that the *Synechocystis* MerA-like protein operates in the protection against, and the NADPH-driven reduction of, mercuric ions [24]. Furthermore, the MerA-like enzyme is also capable to reduce uranyl ions and protect cells, thereby challenging the notion of metal selectivity [24]. Such cyanobacterial enzymes like MerA, with the capacity to reduce HgII and U(VI), and possibly other heavy metal ions, are of interest for future utilization of cyanobacteria for biodetection and/or bioremediation purposes [35], since most polluted sites contain cocktails of toxic metals.

## 6. Glutathione Protects Cells against Oxidative and Metal Stresses

Glutathione, the highly abundant (1–10 mM) tripeptide present in a wealth of organisms from cyanobacteria to eukaryotes, plays a central role in protection against oxidative stress [14,59]. The reduced (major) form of glutathione (hereafter GSH) maintains the intracellular cell environment in a reduced state. GSH serves as an electron donor to the anti-oxidant glutaredoxin enzymes (see below). After oxidation, the resulting glutathione disulfide (GSSG) is regenerated into GSH by various factors, including the NADPH-using enzyme glutathione reductase occurring in many, but not all organisms [60].

Furthermore, GSH also operates in protection against metal stresses. For instance, yeast challenged with arsenite accumulates GSH outside the cells where it chelates As, thereby protecting cells from As toxicity [61]. GSH is also a key component of the cytoplasmic pool of labile iron (Fe), mostly occurring under the Fe(II)-GSH complex, which likely supplies Fe for the synthesis of Fe or [Fe–S] cluster cofactors of metallo-enzymes [62]. Furthermore, GSH operates in the assembly of the [Fe–S] cluster of the anti-oxidant enzymes glutaredoxins (see below) and Fe homeostasis, and GSH play a crucial role in cyanobacterial defenses against oxidative and metal stresses [38,63]. It is not surprising that the crosstalk between GSH, Fe homeostasis and responses to anti-oxidant and metal stresses is important in cyanobacteria because these organisms possess abundant metal-requiring, oxidative-stress generating, machineries for photosynthesis, respiration and nitrogen assimilation [27].

## 7. The Glutaredoxin Enzymes Operate in the Protection against Oxidative and Metal Stresses

As mentioned above, cyanobacteria are continuously challenged with toxic reactive oxygen species generated by photosynthesis and respiration, which can oxidize the thiol group (SH) of two cysteinyl residues to form disulfide bonds (–S–S–) between proteins, or between a protein and a molecule of the crucial [63] anti-oxidant tripeptide glutathione (glutathionylation, Figure 1) [14,22]. The widely conserved glutaredoxin enzymes (Grxs) use electrons provided by either GSH, the thioredoxin reductase enzyme or other Grxs [60,64], to reduce the oxidative-stress-generated disulfides between proteins or glutathione–protein mixed disulfides (glutathionylation), which otherwise affect protein activity and/or stability [65]. The Grxs proteins comprise two main families. The dithiol Grxs, which possess a CX_2_C redox center (C stands for cysteine and X for any other amino acids), catalyze the reduction of protein disulfides or GSH–protein mixed disulfides. The monothiol Grxs, which have a CX_2_S redox active center (S stands for serine), operate in the sensing of cellular iron and in the biogenesis of iron-sulfur clusters of electron-transfer proteins [66]. Some monothiol Grxs have also been reported to catalyze protein deglutathionylation [67]. The poorly understood cyanobacterial Grxs are studied in *Synechocystis* PCC6803 because it possesses only three Grxs, which are all are dispensable to cell growth under standard photoautotrophic conditions [14,68]. The dithiol enzymes Grx1 (Slr1562, also known as GrxB) and Grx2 (Ssr2061, also known as GrxA) operate in the tolerance to arsenate [54,55]. Using a monothiol mutant of Grx2 as the prey protein, 42 Grx2-interacting bait proteins were identified, some of which being involved in tolerance to oxidative stress [69]. Thirteen Grx2 targets, such as the catalase-peroxidase and a peroxyredoxin, which were also identified as Trx partner proteins [70]. Emphasizing on the crosstalk between the Grx and Trx systems, we found that Grx1 and Grx2 operate in an integrative redox pathway, NAD(P)H-thioredoxin reductase–Grx1–Grx2–ferredoxin 7, which transfer electrons in that order, to reduce selenate [60]. The Grx2 enzyme also operates in the tolerance to H_2_O_2_ [60,68] and heat shock [68].

Concerning the selectivity/redundancy of Grxs, we found that Grx1, but neither Grx2 nor Grx3, physically interacts with the above-mentioned mercuric/uranyl reductase enzyme MerA. Furthermore, we showed that the activity of MerA can be inhibited by glutathionylation, and subsequently reactivated by Grx1, likely through deglutathionylation. Consistently, Grx1 appeared to be crucial for the protection against both mercury and uranium, like MerA [24]. Together with other data [23], these findings emphasize the evolutionary conservation of the glutathionylation/deglutathionylation control of enzyme activity, a process mostly described in eukaryotes, so far [64,67].

Concerning the Grx3 monothiol enzyme we showed that it forms a homodimer bridged by a GSH-ligated (2Fe–2S) cluster, a feature conserved in Grx3 orthologs from cyanobacteria to plants and mammals [71,72]. These findings were confirmed by other groups [66,73,74].

## 8. The Ferredoxin Enzymes Are Involved in the Tolerance to Oxidative and Metal Stresses

A large number of proteins, estimated to be about 5% of all proteins in *E. coli*, require an iron-sulfur cluster to be active [26], such as ferredoxins (Fed). The Fed proteins are small, widely conserved proteins, which use an iron–sulfur cluster ([2Fe–2S]; [3Fe–4S] and [4Fe–4S]) to distribute electrons to various metabolic pathways. *Synechocystis* possesses nine ferredoxin-encoding genes designated as *fed1* (*ssl0020*), *fed2* (*sll1382*), *fed3* (*slr1828*), *fed4* (*slr0150*), *fed5* (*slr0148*), *fed6* (*ssl2559*), *fed7* (*sll0662*), *fed8* (*ssr3184*) and *fed9* (*slr2059*). The *fed1-6* genes code for [2Fe-2S] ferredoxins; *fed7* encodes a [4Fe–4S] protein; *fed8* codes for a [3Fe–4S][4Fe–4S] Fed and *fed9* for a [4Fe–4S][4Fe–4S] Fed. In agreement with the pivotal role of Feds in electron transfers, all nine *Synechocystis fed* genes appeared to be highly conserved in cyanobacteria [75]. The *Synechocystis fed* genes are regulated by light fluence and metal availabilities (Fe and Zn), as well as by H_2_O_2_ and Cd [75].

The *fed1*, *fed2*, *fed3*, *fed6* and *fed8* genes appeared to be essential to the photoautotrophic growth of *Synechocystis* whereas the *fed4*, *fed5*, *fed7* and *fed9* are dispensable [10,60,75,76,77]. Interestingly, the absence of Fed7 or Fed9 (but not Fed4 or Fed5) decrease the tolerance to oxidative (H_2_O_2_) and metal stresses (Cd for Fed7; and As and U for Fed9 [75]).

## 9. The Fur-Like Regulators Play a Crucial Role in Metal Homeostasis and the Tolerance to Oxidative Stress and Heavy Metals

Although iron is the fourth most plentiful element in the Earth’s crust, it is frequently a growth-limiting nutrient, because the oxygenic photosynthesis, which emerged in cyanobacteria more than 2.5 billion years ago, raised the oxygen levels that oxidized the soluble ferrous ions (Fe^2+^) to insoluble ferric ions (Fe^3+^) [1,78]. Bacterial cells utilize multiple strategies to maintain their iron homeostasis. These processes include (i) synthesis, export and re-import of powerful ferric ion chelators called siderophores; (ii) dedicated uptake systems; (iii) sequestration in intracellular stores (ferritins); and (iv) degradation of iron-containing proteins in response to Fe-starvation, and subsequent incorporation of the released Fe atoms into crucial Fe-requiring enzymes [27]. These processes are controlled by the widespread Fe-containing ferric uptake regulator (FUR). In the presence of Fe, FUR acts as a transcriptional repressor through binding to AT-rich DNA elements (Fur-boxes) present in the core promoters of Fe-regulated genes. At low Fe concentrations Fe-free FUR (apo-FUR) detaches itself off FUR-boxes, thereby allowing transcription of the downstream genes [27]. FUR also plays a central role in the coordination of the oxidative stress defenses in the cell through as yet unclear mechanisms [79]. The protection against oxidative stress is also regulated by the well-conserved FUR-like protein PerR (peroxide regulator). This PerR protein senses H_2_O_2_ through the Fe-catalyzed oxidation of some of its histidine amino acid residues, leading to dissociation of the PerR-DNA complex. The other (less) conserved FUR-like zinc uptake regulator (ZUR) is mainly involved in repressing the transcription of Zn uptake genes to maintain Zn homeostasis [79].

Metal homeostasis processes are especially important in cyanobacteria because they perform the two main metal-utilizing oxidant-generating processes, respiration and photosynthesis, and the latter iron-rich machinery imposes strong Fe requirements [27,79]. Consistently, *Synechocystis* harbours the three FUR-like regulators. The first FUR-like protein, often designated as FurA (Sll0567), is a likely the true functional FUR homologue. Recently, the *furA* gene appeared to be down-regulated by the PfsR (Sll1392) autoregulator, which operates in the control of Fe homeostasis and light tolerance, emphasizing on the crosstalk between these biological processes [80]. Furthermore, FurA protein-abundance is negatively controlled by the proteolytic FtsH1/FtsH3 heterocomplex located in the cytoplasmic membrane of *Synechocystis* [81].

The second FUR-like regulator, Slr1738, resembles the bacterial peroxide-response regulator PerR in operating in the tolerance to H_2_O_2_ [38,82,83,84]. Slr1738 also plays a crucial role in the tolerance to cadmium, which is intensively spread out in environment as a by-product of Zn-mining, the burning of fossil fuel, the dispersal of sewage sludge, and the manufacturing of paints, batteries and screens [85]. We showed that *Synechocystis* responds to the Cd stress in a two main temporal phases process. In the “early” phase, cells mainly limit Cd entry through the regulation of genes involved in metal uptake and export. Later, the number of responsive genes drastically increases. In this “massive-response” phase, Cd down-regulates most genes operating in: (i) photosynthesis (PS), which normally provides ATP and NADPH; (ii) nutrient (carbon, nitrogen and sulfur) assimilation, which requires ATP and NAD(P)H; and (iii) protein synthesis, a major consumer of ATP and nutrients. Simultaneously, Cd up-regulates numerous genes involved in degradation of abundant PS proteins, thereby liberating Fe and organic carbon and nitrogen for the synthesis of Cd-tolerance proteins. Consistently, Cd also increases expression of the *suf* genes involved in iron-sulfur cluster biogenesis and repair. Collectively, these data suggest that Cd-challenged cells trigger an integrated reprogramming of their whole metabolism, in which the ATP- and nutrients-sparing down-regulation of anabolism limits the poisoning incorporation of Cd into metalloenzymes, while the PS breakdown liberates nutrient assimilates for the synthesis of Cd-tolerance proteins. The most striking common effect of Cd and H_2_O_2_ is the disturbance of light tolerance and Fe homeostasis, which appeared to be interdependent. Also interestingly, our results indicated that cells challenged with H_2_O_2_ or Cd use different strategies for the same purpose of supplying Fe atoms for the synthesis and repair of Fe-requiring metalloenzymes. While H_2_O_2_-challenged cells preferentially accelerate the intake of Fe from the medium, Cd-stressed cells preferentially breakdown the Fe-rich PS machinery to liberate Fe atoms for Fe-requiring enzymes [38].

Finally, the third FUR-like regulator, Sll1937, is a Zn-uptake regulator, Zur [79].

## 10. Conclusions

It is important to characterize the defenses against oxidative and metal stresses in cyanobacteria because they are the organisms that developed most of these mechanisms as a crucial necessity to cope with the production of ROS (reactive oxygen species) by their active metals-requiring photoautotrophic metabolism, which is crucial to the Biosphere in producing a large part of the oxygen and biomass for the food chain. Furthermore, many of the effective anti-oxidant processes, which emerged in cyanobacteria, have been conserved and diversified in higher plants and mammals. In the past few years, significant progress has been made in our understanding of ROS-scavenging and detoxification processes in cyanobacteria. In addition, metal sensors, transporters and stores have been characterized and appeared to also be involved in defenses against oxidative stresses, and *vice versa*. Hence, the crosstalk between the response to oxidative and metal stresses in cyanobacteria is increasingly emerging as crucial to the growth and survival of these environmentally important microorganisms, which also have valuable biotechnological interests, including the carbon-neutral production of biofuels.

## References

[1] Schopf W.J. (2011). The paleobiological record of photosynthesis. Photosynth. Res..

[2] Archibald J.M. (2009). The puzzle of plastid evolution. Curr. Biol..

[3] Jansson C., Northen T. (2010). Calcifying cyanobacteria—The potential of biomineralization for carbon capture and storage. Curr. Opin. Biotechnol..

[4] Rosgaard L., de Porcellinis A.J., Jacobsen J.H., Frigaard N.U., Sakuragi Y. (2012). Bioengineering of carbon fixation, biofuels, and biochemicals in cyanobacteria and plants. J. Biotechnol..

[5] Cassier-Chauvat C., Veaudor T., Chauvat F. (2014). Advances in the function and regulation of hydrogenase in the cyanobacterium *Synechocystis* PCC6803. Int. J. Mol. Sci..

[6] Nakamura Y., Kaneko T., Hirosawa M., Miyajima N., Tabata S. (1998). CyanoBase, a www database containing the complete nucleotide sequence of the genome of *Synechocystis* sp. strain PCC6803. Nucleic Acids Res..

[7] Grigorieva G., Shestakov S. (1982). Transformation in the cyanobacterium *Synechocystis* sp 6803. FEMS Microbiol. Lett..

[8] Marraccini P., Bulteau S., Cassier-Chauvat C., Mermet-Bouvier P., Chauvat F. (1993). A conjugative plasmid vector for promoter analysis in several cyanobacteria of the genera *Synechococcus* and *Synechocystis*. Plant Mol. Biol..

[9] Mermet-Bouvier P., Chauvat F. (1994). A conditional expression vector for the cyanobacteria *Synechocystis* sp. PCC6803 and PCC6714 or *Synechococcus* sp. PCC7942 and PCC6301. Curr. Microbiol..

[10] Poncelet M., Cassier-Chauvat C., Leschelle X., Bottin H., Chauvat F. (1998). Targeted deletion and mutational analysis of the essential [2Fe–2S] plant-like ferredoxin in *Synechocystis* PCC6803 by plasmid shuffling. Mol. Microbiol..

[11] Ortega-Ramos M., Jittawuttipoka T., Saenkham P., Czarnecka-Kwasiborski A., Bottin H., Cassier-Chauvat C., Chauvat F. (2014). Engineering *Synechocystis* PCC6803 for hydrogen production: Influence on the tolerance to oxidative and sugar stresses. PLoS One.

[12] Hess W.R. (2011). Cyanobacterial genomics for ecology and biotechnology. Curr. Opin. Microbiol..

[13] Shih P.M., Wu D., Latifi A., Axen S.D., Fewer D.P., Talla E., Calteau A., Cai F., Tandeau de Marsac N., Rippka R. (2013). Improving the coverage of the cyanobacterial phylum using diversity-driven genome sequencing. Proc. Natl. Acad. Sci. USA.

[14] Narainsamy K., Marteyn B., Sakr S., Cassier-Chauvat C., Chauvat F., Chauvat F., Cassier-Chauvat C. (2013). Genomics of the pleïotropic glutathione system in cyanobacteria. Advances in Botanical Research.

[15] Chauvat F., de Vries L., van der Ende A., van Arkel G.A. (1986). A host-vector system for gene cloning in the cyanobacterium *Synechocystis* PCC6803. Mol. Gen. Genet..

[16] Hagemann M., Chauvat F., Cassier-Chauvat C. (2013). Genomics of salt acclimation: Synthesis of compatible solutes among cyanobacteria. Advances in Botanical Research.

[17] Kirilovsky D., Kerfeld C.A., Chauvat F., Cassier-Chauvat C. (2013). Structural, mechanistic and genomic insights into OCP-mediated photoprotection. Advances in Botanical Research.

[18] Mejean A., Ploux O., Chauvat F., Cassier-Chauvat C. (2013). A genomic view of secondary metabolite production in cyanobacteria. Advances in Botanical Research.

[19] Cassier-Chauvat C., Chauvat F., Flores E., Herrero A. (2014). Cell division in cyanobacteria. The Cell Biology of Cyanobacteria.

[20] Maldener I., Summers M.L., Sukenik A., Flores E., Herrero A. (2014). Cellular differentiation in filamentous cyanobacteria. The Cell Biology of Cyanobacteria.

[21] Narainsamy K., Cassier-Chauvat C., Junot C., Chauvat F. (2011). High performance analysis of the cyanobacterial metabolism via liquid chromatography coupled to a LTQ-orbitrap mass spectrometer: Evidence that glucose reprograms the whole carbon metabolism and triggers oxidative stress. Metabolomics.

[22] Imlay J.A. (2013). The molecular mechanisms and physiological consequences of oxidative stress: Lessons from a model bacterium. Nat. Rev. Microbiol..

[23] Chardonnet S., Sakr S., Cassier-Chauvat C., le Marechal P., Chauvat F., Lemaire S.D., Decottignies P. (2014). First proteomic study of *S*-glutathionylation in cyanobacteria. J. Proteome Res..

[24] Marteyn B., Sakr S., Farci S., Bedhomme M., Chardonnet S., Decottignies P., Lemaire S.D., Cassier-Chauvat C., Chauvat F. (2013). The *Synechocystis* PCC6803 MerA-like enzyme operates in the reduction of both mercury and uranium under the control of the glutaredoxin 1 enzyme. J. Bacteriol..

[25] Sakr S., Dutheil J., Saenkham P., Bottin H., Leplat C., Ortega-Ramos M., Aude J.C., Chapuis V., Guedeney G., Decottignies P. (2013). The activity of the *Synechocystis* PCC6803 AbrB2 regulator of hydrogen production can be post-translationally controlled through glutathionylation. Int. J. Hydrog. Energy.

[26] Waldron K.J., Robinson N.J. (2009). How do bacterial cells ensure that metalloproteins get the correct metal?. Nat. Rev. Microbiol..

[27] Kranzler C., Rudolf M., Keren N., Schleiff E. (2013). Iron in cyanobacteria. Adv. Bot. Res..

[28] Blindauer C.A. (2008). Zinc-handling in cyanobacteria: An update. Chem. Biodivers..

[29] Tchounwou P.B., Yedjou C.G., Patlolla A.K., Sutton D.J. (2012). Heavy metal toxicity and the environment. Mol. Clin. Environ. Toxicol..

[30] Song Q., Li J. (2014). Environmental effects of heavy metals derived from the e-waste recycling activities in China: A systematic review. Waste Manag..

[31] Chetelat J., Amyot M., Arp P., Blais J.M., Depew D., Emmerton C.A., Evans M., Gamberg M., Gantner N., Girard C. (2014). Mercury in freshwater ecosystems of the Canadian Arctic: Recent advances on its cycling and fate. Sci. Total Environ..

[32] Gregoire D.S., Poulain A.J. (2014). A little bit of light goes a long way: The role of phototrophs on mercury cycling. Metallomics.

[33] Imlay J.A. (2014). The Mismetallation of enzymes during oxidative stress. J. Biol. Chem..

[34] Heindl J.E., Wang Y., Heckel B.C., Mohari B., Feirer N., Fuqua C. (2014). Mechanisms and regulation of surface interactions and biofilm formation in *Agrobacterium*. Front. Plant Sci..

[35] De Philippis R., Colica G., Micheletti E. (2011). Exopolysaccharide-producing cyanobacteria in heavy metal removal from water: Molecular basis and practical applicability of the biosorption process. Appl. Microbiol. Biotechnol..

[36] Jittawuttipoka T., Planchon M., Spalla O., Benzerara K., Guyot F., Cassier-Chauvat C., Chauvat F. (2013). Multidisciplinary evidences that *Synechocystis* PCC6803 exopolysaccharides operate in cell sedimentation and protection against salt and metal stresses. PLoS One.

[37] Planchon M., Jittawuttipoka T., Cassier-Chauvat C., Guyot F., Chauvat F., Spalla O. (2013). Influence of exopolysaccharides on the electrophoretic properties of the model cyanobacterium *Synechocystis*. Colloids Surf..

[38] Houot L., Floutier M., Marteyn B., Michaut M., Picciocchi A., Legrain P., Aude J.C., Cassier-Chauvat C., Chauvat F. (2007). Cadmium triggers an integrated reprogramming of the metabolism of *Synechocystis* PCC6803, under the control of the Slr1738 regulator. BMC Genomics.

[39] Barras F., Fontecave M. (2011). Cobalt stress in *Escherichia coli* and *Salmonella enterica*: Molecular bases for toxicity and resistance. Metallomics.

[40] Zeyons O., Thill A., Chauvat F., Menguy N., Cassier-Chauvat C., Orear C., Daraspe J., Auffan M., Rose J., Spalla O. (2009). Direct and indirect CeO_2_ nanoparticles toxicity for *Escherichia coli* and *Synechocystis*. Nanotoxicology.

[41] Planchon M., Jittawuttipoka T., Cassier-Chauvat C., Guyot F., Gelabert A., Benedetti M.F., Chauvat F., Spalla O. (2013). Exopolysaccharides protect *Synechocystis* against the deleterious effects of Titanium dioxide nanoparticles in natural and artificial waters. J. Colloid Interface Sci..

[42] Von Moos N., Slaveykova V.I. (2014). Oxidative stress induced by inorganic nanoparticles in bacteria and aquatic microalgae—State of the art and knowledge gaps. Nanotoxicology.

[43] Turner J.S., Morby A.P., Whitton B.A., Gupta A., Robinson N.J. (1993). Construction of Zn^2+^/Cd^2+^ hypersensitive cyanobacterial mutants lacking a functional metallothionein locus. J. Biol. Chem..

[44] Blindauer C.A. (2011). Bacterial metallothioneins: Past, present, and questions for the future. J. Biol. Inorg. Chem..

[45] Huckle J.W., Morby A.P., Turner J.S., Robinson N.J. (1993). Isolation of a prokaryotic metallothionein locus and analysis of transcriptional control by trace-metal ions. Mol. Microbiol..

[46] Morby A.P., Turner J.S., Huckle J.W., Robinson N.J. (1993). SmtB is a metal-dependent repressor of the cyanobacterial metallothionein gene *smtA*—Identification of a Zn inhibited DNA-protein complex. Nucleic Acids Res..

[47] Thelwell C., Robinson N.J., Turner-Cavet J.S. (1998). An SmtB-like repressor from *Synechocystis* PCC6803 regulates a zinc exporter. Proc. Natl. Acad. Sci. USA.

[48] Garcia-Dominguez M., Lopez-Maury L., Florencio F.J., Reyes J.C. (2000). A gene cluster involved in metal homeostasis in the cyanobacterium *Synechocystis* sp. strain PCC6803. J. Bacteriol..

[49] Chen L., Zhu Y., Song Z., Wang J., Zhang W. (2014). An orphan response regulator Sll0649 involved in cadmium tolerance and metal homeostasis in photosynthetic *Synechocystis* sp. PCC6803. J. Proteomics.

[50] Nagy C.I., Vass I., Rakhely G., Vass I.Z., Toth A., Duzs A., Peca L., Kruk J., Kos P.B. (2014). Coregulated genes link sulfide:quinone oxidoreductase and arsenic metabolism in *Synechocystis* sp. strain PCC6803. J. Bacteriol..

[51] Sanchez-Riego A.M., Lopez-Maury L., Florencio F.J. (2014). Genomic responses to arsenic in the cyanobacterium *Synechocystis* sp. PCC6803. PLoS One.

[52] Lopez-Maury L., Florencio F.J., Reyes J.C. (2003). Arsenic sensing and resistance system in the cyanobacterium *Synechocystis* sp. strain PCC6803. J. Bacteriol..

[53] Li R., Haile J.D., Kennelly P.J. (2003). An arsenate reductase from *Synechocystis* sp. strain PCC6803 exhibits a novel combination of catalytic characteristics. J. Bacteriol..

[54] Kim S.G., Chung J.S., Sutton R.B., Lee J.S., Lopez-Maury L., Lee S.Y., Florencio F.J., Lin T., Zabet-Moghaddam M., Wood M.J. (2012). Redox, mutagenic and structural studies of the glutaredoxin/arsenate reductase couple from the cyanobacterium *Synechocystis* sp. PCC6803. Biochim. Biophys. Acta.

[55] Lopez-Maury L., Sanchez-Riego A.M., Reyes J.C., Florencio F.J. (2009). The glutathione/glutaredoxin system is essential for arsenate reduction in *Synechocystis* sp. strain PCC6803. J. Bacteriol..

[56] Yin X.X., Chen J., Qin J., Sun G.X., Rosen B.P., Zhu Y.G. (2011). Biotransformation and volatilization of arsenic by three photosynthetic cyanobacteria. Plant Physiol..

[57] Wall J.D., Krumholz L.R. (2006). Uranium reduction. Ann. Rev. Microbiol..

[58] Lefebvre D.D., Kelly D., Budd K. (2007). Biotransformation of Hg(II) by cyanobacteria. Appl. Environ. Microbiol..

[59] Cameron J.C., Pakrasi H.B. (2011). Glutathione in *Synechocystis* 6803: A closer look into the physiology of a *ΔgshB* mutant. Plant Signal. Behav..

[60] Marteyn B., Domain F., Legrain P., Chauvat F., Cassier-Chauvat C. (2009). The thioredoxin reductase-glutaredoxins-ferredoxin crossroad pathway for selenate tolerance in *Synechocystis* PCC6803. Mol. Microbiol..

[61] Thorsen M., Jacobson T., Vooijs R., Navarrete C., Bliek T., Schat H., Tamas M.J. (2012). Glutathione serves an extracellular defence function to decrease arsenite accumulation and toxicity in yeast. Mol. Microbiol..

[62] Hider R.C., Kong X.L. (2011). Glutathione: A key component of the cytoplasmic labile iron pool. Biometals.

[63] Cameron J.C., Pakrasi H.B. (2010). Essential role of glutathione in acclimation to environmental and redox perturbations in the cyanobacterium *Synechocystis* sp. PCC6803. Plant Physiol..

[64] Zaffagnini M., Bedhomme M., Marchand C.H., Morisse S., Trost P., Lemaire S.D. (2012). Redox regulation in photosynthetic organisms: Focus on glutathionylation. Antioxid. Redox Signal..

[65] Lillig C.H., Berndt C. (2012). Glutaredoxins in thiol/disulfide exchange. Antioxid. Redox Signal..

[66] Rouhier N., Couturier J., Johnson M.K., Jacquot J.P. (2010). Glutaredoxins: Roles in iron homeostasis. Trends Biochem. Sci..

[67] Zaffagnini M., Bedhomme M., Lemaire S.D., Trost P. (2012). The emerging roles of protein glutathionylation in chloroplasts. Plant Sci..

[68] Sanchez-Riego A.M., Lopez-Maury L., Florencio F.J. (2013). Glutaredoxins are essential for stress adaptation in the cyanobacterium *Synechocystis* sp. PCC6803. Front. Plant Sci..

[69] Li M., Yang Q., Zhang L., Li H., Cui Y., Wu Q. (2007). Identification of novel targets of cyanobacterial glutaredoxin. Arch. Biochem. Biophys..

[70] Florencio F.J., Perez-Perez M.E., Lopez-Maury L., Mata-Cabana A., Lindahl M. (2006). The diversity and complexity of the cyanobacterial thioredoxin systems. Photosynth. Res..

[71] Picciocchi A., Saguez C., Boussac A., Cassier-Chauvat C., Chauvat F. (2007). CGFS-type monothiol glutaredoxins from the cyanobacterium *Synechocystis* PCC6803 and other evolutionary distant model organisms possess a glutathione-ligated [2Fe–2S] cluster. Biochemistry.

[72] Iwema T., Picciocchi A., Traore D.A., Ferrer J.L., Chauvat F., Jacquamet L. (2009). Structural basis for delivery of the intact [Fe2S2] cluster by monothiol glutaredoxin. Biochemistry.

[73] Lillig C.H., Berndt C., Holmgren A. (2008). Glutaredoxin systems. Biochim. Biophys. Acta.

[74] Herrero E., Belli G., Casa C. (2010). Structural and functional diversity of glutaredoxins in yeast. Curr. Protein Pept. Sci..

[75] Cassier-Chauvat C., Chauvat F. (2014). Function and regulation of ferredoxins in the cyanobacterium, *Synechocystis* PCC6803: Recent advances. Life.

[76] Gutekunst K., Chen X., Schreiber K., Kaspar U., Makam S., Appel J. (2014). The bidirectional NiFe-hydrogenase in *Synechocystis* sp. PCC6803 is reduced by flavodoxin and ferredoxin and is essential under mixotrophic, nitrate-limiting conditions. J. Biol. Chem..

[77] Mustila H., Allahverdiyeva Y., Isojarvi J., Aro E.M., Eisenhut M. (2014). The bacterial-type [4Fe–4S] ferredoxin 7 has a regulatory function under photooxidative stress conditions in the cyanobacterium *Synechocystis* sp. PCC6803. Biochim. Biophys. Acta.

[78] Behrenfeld M.J., Milligan A.J. (2013). Photophysiological expressions of iron stress in phytoplankton. Ann. Rev. Mar. Sci..

[79] Fillat M.F. (2014). The FUR (ferric uptake regulator) superfamily: Diversity and versatility of key transcriptional regulators. Arch. Biochem. Biophys..

[80] Cheng D., He Q.F. (2014). PfsR is a key regulator of iron homeostasis in *Synechocystis* PCC6803. PLoS One.

[81] Krynicka V., Tichy M., Krafl J., Yu J., Kana R., Boehm M., Nixon P.J., Komenda J. (2014). Two essential FtsH proteases control the level of the Fur repressor during iron deficiency in the cyanobacterium *Synechocystis* sp. PCC6803. Mol. Microbiol..

[82] Kobayashi M., Ishizuka T., Katayama M., Kanehisa M., Bhattacharyya-Pakrasi M., Pakrasi H.B., Ikeuchi M. (2004). Response to oxidative stress involves a novel peroxiredoxin gene in the unicellular cyanobacterium *Synechocystis* sp. PCC6803. Plant Cell Physiol..

[83] Li H., Singh A.K., McIntyre L.M., Sherman L.A. (2004). Differential gene expression in response to hydrogen peroxide and the putative PerR regulon of *Synechocystis* sp. strain PCC6803. J. Bacteriol..

[84] Garcin P., Delalande O., Zhang J.Y., Cassier-Chauvat C., Chauvat F., Boulard Y. (2012). A transcriptional-switch model for Slr1738-controlled gene expression in the cyanobacterium *Synechocystis*. BMC Struct. Biol..

[85] Cullen J.T., Maldonado M.T. (2013). Biogeochemistry of cadmium and its release to the environment. Met. Ions Life Sci..

